# Epidemiologic Surveillance in an academic hospital during the COVID-19 pandemic in Sao Paulo, Brazil: the key role of epidemiologic engagement in operational processes

**DOI:** 10.6061/clinics/2020/e2166

**Published:** 2020-07-15

**Authors:** Izabel Marcilio, Anna Miethke-Morais, Leila Harima, Amanda C. Montal, Beatriz Perondi, Jose Ricardo de Carvalho Mesquita Ayres, Nelson Gouveia, Eloisa Bonfa, Hillegonda Maria Dutilh Novaes

**Affiliations:** I Hospital das Clinicas HCFMUSP, Faculdade de Medicina, Universidade de Sao Paulo, Sao Paulo, SP, BR; II Departamento de Medicina Preventiva, Faculdade de Medicina FMUSP, Universidade de São Paulo, Sao Paulo, SP, BR

On January 30, 2020, the Director-General of the World Health Organization (WHO) declared the severe acute respiratory syndrome coronavirus 2 (SARS-CoV-2) outbreak a public health emergency of international concern under the International Health Regulations (2005) (1). As an immediate response effort, Hospital das Clínicas da Faculdade de Medicina da USP (HCFMUSP) mobilized its Crisis Management Committee (CMC) to deal with the outbreak by assisting with strategic planning. As stipulated in the CMC’s organogram for outbreak incidents (2), the Epidemiologic Surveillance Center (Núcleo de Vigilância Epidemiológica (NUVE)) was engaged from the beginning in planning, assisting the incident commander by providing event-specific advice during the coronavirus disease (COVID-19) pandemic.

HCFMUSP is a tertiary teaching hospital in Sao Paulo, Brazil, usually dedicated to treating high-complexity cases, and it comprises eight specialized institutes accounting for approximately 2,200 beds and 20,000 healthcare personnel. Its main building operates with about 900 beds and 33 different medical specialties and includes an Emergency Room and an 84-bed intensive care unit (ICU). Beginning in March 2020, the number of severe COVID-19 cases in Sao Paulo started to rise rapidly, and the demand for ICU beds outnumbered the then available resources in the Metropolitan Region of Sao Paulo. In an attempt to meet this demand, the CMC decided to isolate its Central Institute, transferring all non-COVID cases to other institutes or to the Emergency Room. This strategy increased the number of ICU beds devoted exclusively to COVID-19 patients by about 4-fold, to a total of 300 beds. As expected, this unprecedented but necessary transformation affected all sectors within the hospital, including the Epidemiologic Surveillance Center. For instance, while in the past an average of 3200 cases per year were reported to the central level of epidemiologic surveillance, during the pandemic NUVE reported 3300 cases in May 2020 alone.

Epidemiological surveillance is the continuous, systematic collection, analysis, and interpretation of health-related data needed for the planning, implementation, and evaluation of public health practice (1). In Brazil, to increase coverage and effectiveness of the surveillance system and thus reduce sub-notification of cases, the Ministry of Health has implemented the National Hospital Epidemiological Surveillance System, integrating Hospital Surveillance Centers in selected hospitals into a surveillance network. There are currently over 240 of these centers distributed throughout the country, and considering that hospitals are in a privileged position for the early detection and accurate diagnosis of cases, these centers play a strategic role in the epidemiologic surveillance system in Brazil (3).

The Epidemiologic Surveillance Center routinely monitors public information systems for events related to the occurrence of infectious diseases, aiming at the early detection and notification of health events. Epidemiologic surveillance is undertaken to inform decision making on disease prevention and control measures (4). NUVE was responsible for detecting the first patient with suspected COVID-19 hospitalized in the city of Sao Paulo, thus providing an early warning to the CMC of the approaching need to accelerate the initial preventive measures.

Epidemiologic surveillance is key for the response strategy in a pandemic, and surveillance data are essential to calibrate appropriate and proportionate public health measures (1). The COVID-19 pandemic is caused by an agent hitherto unknown to the scientific community that has the capacity to cause a severe disease with a high case-fatality rate. For an Epidemiologic Surveillance Center, working in this environment represented both a unique opportunity to learn and bring resources to the field and a challenge to overcome. As an Epidemiologic Surveillance Center within the main reference hospital for severe COVID-19 cases in the state of Sao Paulo, NUVE had two additional challenges to meet: adapting to an unforeseen escalation in workload related to notification of cases to the central health authority and communicating and providing information at the operational level—that is, information related to problems to be solved by decision-makers in the hospital and, more specifically, by the CMC.

To overcome the first challenge, NUVE adapted its routine processes and workflow. Initially, case definitions and investigation protocols were distributed to health workers in the hospital, in line with the guidance from the Ministry of Health. To standardize and facilitate health data collection for notification of cases and to avoid extra work for the already overburdened health workers, a simplified notification file was added to the electronic medical record. Importantly, NUVE increased its workforce by recruiting and training medical residents from different specialties to carry out epidemiologic investigations to aid in the notification of COVID-19 cases. Furthermore, to enhance case finding, the automatic warning system (Labovigi), created by NUVE and implemented by the IT laboratory team, which sends alerts whenever a positive result for selected laboratory tests is generated, was adapted to include results of SARS-CoV-2 reverse-transcription polymerase chain reactions and serology assays.

We also adapted our case registry database system to collect specific data identified as important information for other sectors in the hospital. For instance, as reports from other countries had shown that infection of health workers is not only common but represents an important hindrance in the response to the pandemic, NUVE’s digital system was adapted to register variables related to the function and workplace of each professional who tested positive, allowing very early detection of cases for immediate adoption of containment measures. To date, 5574 symptomatic health workers have been tested for COVID-19, with 2006 (36%) of them testing positive. Such information was readily and regularly shared with the Hospital’s Infection Control Committee. Of note, NUVE’s database works interoperably with other HCFMUSP electronic systems. Finally, using an open-source statistical software package (R), NUVE produces a daily epidemiologic report that can easily be shared electronically with stakeholders to continuously inform risk assessment and support operational decision-making on the response to COVID-19. Following these implementations, NUVE was able to cope with the massive increase in workload and managed to notify all suspect cases within 24 hours of hospitalization.

With such a large dataset of cases, NUVE has provided both information and datasets to many research groups within the HCFMUSP from very different fields of research. The novelty of the SARS-CoV-2 virus has inspired a wide variety of scientific research worldwide, and the situation would not be different in a teaching hospital. In fact, WHO itself encourages efforts and research to investigate additional epidemiological, virologic, and clinical characteristics of the disease (1). The opportunity to collaborate with some of those groups is a welcome consequence of the pandemic and may be useful for future needs.

The second challenge faced by NUVE as a hospital-based epidemiologic surveillance center while working in the pandemic was to bring epidemiologic information to the operational level, that is, to communicate data in a way that would be helpful to decision-makers. Doing so is even more important in this era of “infodemia,” when we are overwhelmed with the anxious dissemination of a large amount of information, sometimes accurate but often not. It is also an era wherein the constantly increasing computational resources have led many to disregard reasoning as a necessary step in interpreting reality, and “big data” is seen as a solution to problems that we have not yet properly defined. From the epidemiologic surveillance perspective, information is gold and should be analyzed with intelligence and integrity, and data alone are useless unless acted upon. In fact, the many-step process of choosing, collecting, collating, analyzing, and presenting data should interact with the health care planning and decision makers so that the intelligence systems are continually adapted to their needs (5). Thus, since the beginning of HCFMUSP’s response to COVID-19, NUVE has been working in close collaboration with the CMC to provide timely and strategic information.

Epidemiologists are not often engaged in such operational duties, nor are hospital managers acquainted with the daily use of epidemiologic surveillance reports. Nevertheless, the new clinical conditions, diagnosis, and treatment associated with COVID-19 have made it vital to combine these two areas of expertise, and the HCFMUSP experience is a powerful testimony to this practice. This is undoubtedly a challenging goal for every health care team, which is certainly not easily achieved; however, many positive steps have been taken in the right direction, and encouraging results are already being reached.

## AUTHOR CONTRIBUTIONS

All coauthors have provided substantial intellectual contributions to the manuscript and agree with its content and submission.
